# Complete Genome Sequence of *Staphylococcus aureus* XN108, an ST239-MRSA-SCC*mec* III Strain with Intermediate Vancomycin Resistance Isolated in Mainland China

**DOI:** 10.1128/genomeA.00449-14

**Published:** 2014-07-24

**Authors:** Xia Zhang, Xiaomeng Xu, Wenchang Yuan, Qiwen Hu, Weilong Shang, Xiaomei Hu, Yigang Tong, Xiancai Rao

**Affiliations:** a Department of Microbiology, School of Medical Sciences, Third Military Medical University, Chongqing, China; b State Key Laboratory of Pathogen and Biosecurity, Beijing Institute of Microbiology and Epidemiology, Beijing, China

## Abstract

ST239-MRSA-SCC*mec* III (ST239, sequence type 239; MRSA, methicillin-resistant *Staphylococcus aureus*; SCC*mec* III, staphylococcal cassette chromosome *mec* type III) is the most predominant clone of hospital-acquired methicillin-resistant *S. aureus* in mainland China. We report here the complete genome sequence of XN108, the first vancomycin-intermediate *S. aureus* strain isolated from a steam-burned patient with a wound infection.

## GENOME ANNOUNCEMENT

Methicillin-resistant *Staphylococcus aureus* (MRSA) is an important pathogen worldwide. Sequence type 239 and staphylococcal chromosome cassette *mec* element type III MRSA (ST239-MRSA-SCC*mec* III) represents the most prevalent clone that is associated with hospital-acquired infections in mainland China (1, 2). Being reserved as a drug of last resort, the extensive use of vancomycin may lead to the emergence of vancomycin-intermediate *S. aureus* (VISA) isolates. We report here the first complete genome sequence of ST239 VISA isolate XN108, with a vancomycin MIC of 12 µg/ml, recovered from a 34-year-old patient with a wound infection from a steam burn (3).

Whole-genome sequencing was carried out using the Ion Torrent PGM sequencer with a 300-bp single-end library and a 4-kb mate-pair library, which generated 1,042,678 reads (approximately 85× coverage of the genome) and 2,885,240 reads (approximately 116× coverage of the genome), respectively. The resulting sequences were *de novo* assembled in the Roche 454 Newbler 2.5 assembler (4), and 6 primary scaffolds were connected according to the mate-pair relationships. The internal gaps were filled using GapFiller version 1.11 (5). The assembly of the completed genome was validated to be correct by reference to the SmaI restriction map generated by pulsed-field gel electrophoresis. The complete genome of *S. aureus* strain XN108 contains a circular 3,052,055-bp chromosome, with a 32.76% G+C content and a circular plasmid of 31,500 bp in size. The annotations of the chromosome and plasmid were performed with Rapid Annotations using Subsystems Technology (RAST) server (6), followed by manual curation and comparisons with other completed *S. aureus* genomes. A total of 2,992 predicted coding DNA sequences (CDSs), 57 tRNA-coding genes, 15 rRNA operons, and SCC*mec* type III were detected on the chromosome of XN108. More than 89% of the genes were assigned to speciﬁc Clusters of Orthologous Groups (COG) functional groups.

Four genomes of ST239 *S. aureus* strains have been published: JKD6008 (GenBank accession no. CP002120) isolated in New Zealand (7), TW20 (EMBL accession no. FN433596) from England (8), T0131 (GenBank accession no. CP002643) from China (9), and Z172 (GenBank accession no. CP006838) obtained from Taiwan (10). Comparisons with the four *S. aureus* genomes of the ST239 complex revealed that XN108 shares 2,223, 2,401, 2,211, and 2,366 orthologous coding sequences (CDSs) with JKD6008, TW20, T0131, and Z172, respectively. XN108 also has a plasmid of 31.5 kb (pXN108), whereas T0131 contains no extrachromosomal elements, JKD6008 harbors a 28-kb integrated pSK1-like plasmid, TW20 has two plasmids (pTW20_1 and pTW20_2), and Z172 also has two plasmids (pZ172_1 and pZ172_2). The differences in the chromosomes and extrachromosomal elements of *S. aureus* ST239 members may provide evidence of their adaptation to the different continental environments.

### Nucleotide sequence accession number.

This whole-genome sequence of *S. aureus* XN108 has been deposited in NCBI GenBank under the accession no. CP007447.
